# COMT Val158Met and BDNF Val66Met Single-Nucleotide Polymorphisms Are Not Associated With Emotional Distress One Year After Moderate-Severe Traumatic Brain Injury

**DOI:** 10.1089/neur.2023.0028

**Published:** 2023-08-07

**Authors:** Chloe Anderson, Amelia J. Hicks, Jai Carmichael, Richard Burke, Jennie Ponsford

**Affiliations:** ^1^ Monash-Epworth Rehabilitation Research Centre, Epworth HealthCare, Melbourne, Australia; Turner Institute for Brain and Mental Health, School of Psychological Sciences, Monash University, Clayton, Victoria, Australia.; ^2^ School of Biological Sciences, Monash University, Clayton, Victoria, Australia.

**Keywords:** age, BDNF Val66Met, COMT Val158Met, emotional distress, genetics, sex, TBI, traumatic brain injury

## Abstract

Emotional distress is a common, but poorly addressed, feature of moderate-severe traumatic brain injury (TBI). Previously identified sociodemographic, psychological, and injury-related factors account for only a small proportion of the variability in emotional distress post-TBI. Genetic factors may help to further understand emotional distress in this population. The catechol-*O*-methyltransferase (COMT) Val158 and brain-derived neurotrophic factor (BDNF) 66Met single-nucleotide polymorphisms (SNPs) have been identified as possible contributory factors to outcomes after TBI. We investigated whether the COMT Val158 and BDNF 66Met SNPs were associated with emotional distress 1 year after moderate-severe TBI, and whether these associations were moderated by age, sex, and TBI severity (as measured by the duration of post-traumatic amnesia [PTA]). Moderate-severe TBI survivors (COMT, *n* = 391; BDNF, *n* = 311) provided saliva samples after admission to a TBI rehabilitation hospital. At a follow-up interview ∼1 year after injury, participants completed a self-report measure of emotional distress (Hospital Anxiety and Depression Scale; HADS). Multiple linear regression models were constructed for each SNP to predict total scores on the HADS. Neither COMT Val158 nor BDNF 66Met carriage status (carrier vs. non-carrier) significantly predicted emotional distress (COMT, *p* = 0.49; BDNF, *p* = 0.66). Interactions of SNP × age (COMT, *p* = 0.90; BDNF, *p* = 0.93), SNP × sex (COMT, *p* = 0.09; BDNF, *p* = 0.60), SNP × injury severity (COMT, *p* = 0.53; BDNF, *p* = 0.87), and SNP × sex × age (COMT, *p* = 0.08; BDNF, *p* = 0.76) were also non-significant. Our null findings suggest that COMT Val158 and BDNF 66Met SNPs do not aid the prediction of emotional distress 1 year after moderate-severe TBI, neither in isolation nor in interaction with age, sex and injury severity. The reporting of null findings such as ours is important to avoid publication bias and prompt researchers to consider the challenges of single-gene candidate studies in understanding post-TBI outcomes. Analyses in larger samples that incorporate multiple genetic factors and their relevant moderating factors may provide a greater understanding of the role of genetics in post-TBI emotional distress.

## Introduction

Emotional distress is common after moderate-severe traumatic brain injury (TBI),^1,2^ with the prevalence of post-TBI anxiety and depressive disorders estimated at 36% and 43%, respectively.^3^ Emotional distress post-TBI usually peaks in the first year post-injury. The causes of elevated emotional distress after moderate-severe TBI are likely to be multi-factorial, including injury to brain structures that are involved in emotional regulation, as well as a psychological response to post-injury functional limitations.^2^

Not all TBI survivors, however, experience ongoing emotional distress after their injury. Risk factors identified in previous studies include pre-injury mental health problems, comorbid limb injury, history of a previous TBI, avoidant coping and emotion focused coping mechanisms, earlier time post-injury, middle age at injury (∼30–60 years old), female sex (mixed findings), and TBI severity (mixed findings).^3–9^ These psychological, sociodemographic, and injury-related factors do not, however, account for the heterogeneity in post-TBI emotional distress, even when examined in combination.^7^ Therefore, investigating other risk factors for post-TBI emotional distress is important for identifying persons who may be at risk of emotional distress after TBI. Genetic factors could also contribute to post-TBI emotional distress, through modulating the extent of neurotrauma, neural repair, and plasticity, impacting pre-injury personality traits, and affecting neurocognitive and behavioral reserve (i.e., the brain's resistance to neurocognitive and behavioral change subsequent to damage).^10^ Two highly researched genetic factors that may contribute to outcomes after TBI are the catechol-*O*-methyltransferase (COMT) Val158Met and brain-derived neurotrophic factor (BDNF) Val66Met single-nucleotide polymorphisms (SNPs).

The COMT gene encodes an enzyme that breaks down catecholamine neurotransmitters (e.g., dopamine) and is a significant determinant of pre-frontal cortex dopamine concentration.^11,12^ The Val158 SNP of the COMT gene results in increased activity in the enzyme and therefore lower levels of dopamine in the pre-frontal cortex.^13^ In the early stages after a TBI, the expression of catecholamines (and therefore dopamine concentration) in the pre-frontal cortex often decreases, likely attributable to damage in regions of the brain that modulate and transport dopamine.^14^ The pre-frontal cortex plays a critical role in the regulation of emotions, by exerting top-down control of chemical signaling and connectivity in subcortical regions such as the striatum (reward processing), amygdala (emotional processing), and cingulate cortex (emotional and behavioral processing).^15^ Psychiatric disorders, such as anxiety and depression, are commonly linked to dysregulated or decreased dopamine concentrations in the pre-frontal cortex.^16–19^ For TBI survivors carrying the COMT Val158 SNP, dopamine concentration in these subregions may be significantly reduced compared to non-carriers, because of the enzyme's increased activity. Carriage of COMT Val158 could therefore increase the risk of psychiatric illness in TBI survivors.

Only two studies have directly investigated the association of COMT Val158 with emotional distress post-TBI, with conflicting results. One study found that COMT Val158 was significantly associated with a higher incidence of post-traumatic stress disorder 6 months after mild TBI.^12^ Conversely, COMT Val158 was not a significant predictor of depression 1 year after severe TBI.^20^ The association of COMT Val158 with emotional distress after moderate-severe TBI remains unclear and requires further investigation.

The BDNF gene encodes proteins that regulate neuronal plasticity, survival, and apoptosis, particularly in the pre-frontal cortex and hippocampus.^21,22^ BDNF is first synthesized as “pro-BDNF,” which may then be cleaved into “mature BDNF,” which supports plasticity, growth, and survival.^23^ If pro-BDNF is not cleaved, however, it acts in opposition to the mature protein, promoting apoptosis and reducing plasticity.^24^ The BDNF 66Met SNP disrupts the intracellular processing of pro-BDNF, leading to decreased concentrations of both pro-BDNF and mature BDNF in the pre-frontal cortex.^25^ In non-TBI populations, BDNF 66Met has been linked with increased reporting of anxiety-related avoidance behaviors^26^ and higher self-reported depression in adults.^27^ The role of BDNF in neuronal repair and neuroplasticity in brain regions underlying emotional function could mean that carrying the BDNF 66Met SNP is disadvantageous for TBI survivors, possibly increasing the risk of experiencing emotional distress.

The association between BDNF genotype and emotional distress after TBI has only been investigated in mild TBI samples to date. One group found a significant association between the BDNF 66Met SNP and self-reported depression at 1, but not 6, weeks after mild TBI.^28^ Conversely, the SNP was found to be advantageous to psychological functioning and quality-of-life outcomes in children after mild TBI.^29,30^ Lastly, in a separate mild TBI sample of adults, BDNF 66Met was not significantly associated with depression symptoms.^31^ The role of the BDNF 66Met SNP in emotional outcomes after moderate-severe TBI has not been investigated.

Inconsistent findings regarding the associations of the COMT Val158 and BDNF 66Met SNPs with post-TBI emotional distress may reflect a number of factors. First, previous samples have comprised mostly mild TBI survivors. Moderate-severe TBI results in more extensive neuropathological changes^32,33^ and is usually associated with more enduring emotional distress.^32^ COMT and BDNF genotypes may contribute differently to outcomes after moderate-severe TBI as compared to mild TBI, because of their roles in neural repair and plasticity. Second, the measures used in previous studies to assess emotional distress often document common TBI-related symptoms, such as fatigue and concentration difficulties, possibly confounding results.^33^ For example, the Hospital Anxiety and Depression Scale (HADS), a 14-item self-report questionnaire, has been validated in TBI samples as a sound summary measure of emotional distress, and minimizes the inclusion of the items that overlap with commonly occurring symptoms of TBI such as cognitive problems and sleep disturbances.^34,35^ Third, the associations of single-candidate genes with a polygenic trait like emotional distress may be modest, and previous samples (COMT, *n* = 90–93; BDNF, *n* = 52–219) may have been too small to detect these effects with significant statistical power. Fourth, inconsistent findings in the literature may have resulted from a failure to consider potential moderating variables.

A recent review by our research group identified that associations between genetic factors, including COMT Val158 and BDNF 66Met, and outcomes after TBI may vary as a function of other factors such as age, biological sex, and injury severity.^36^ Further, we recently demonstrated the utility of accounting for potential moderating factors on gene-outcome associations after TBI in a study of the association between the apolipoprotein E (APOE) ɛ4 SNP and emotional distress. We found that the contribution of the APOE ɛ4 SNP to post-TBI emotional distress varied across the age of the participants, representing a risk factor only among older TBI survivors.^37^ Similarly, regarding the COMT Val158 and BDNF 66Met SNPs, the literature has provided some evidence that these genetic factors may be associated with TBI outcomes in an age-dependent manner, potentially attributable to changes in the dopaminergic system and concentrations of BDNF target receptors that occur across the life span.^31,38–41^ It is possible that biological sex also moderates the associations of the COMT Val158 and BDNF 66Met SNPs with emotional distress, given that the expression of both SNPs are impacted by sex hormones.^20,28,36^ Lastly, TBI severity may moderate these gene-outcome associations, because of the involvement of these genes in neural repair and recovery in brain areas important for emotional regulation.

In the current study, we investigated whether the COMT Val158 and BDNF 66Met SNPs were associated with emotional distress 1 year after moderate-severe TBI. Additionally, we explored whether any associations between these SNPs and emotional distress were moderated by age, sex, and TBI severity. We formed the following hypotheses:

1. Adults with moderate-severe TBI carrying one or more copies of the COMT Val158 SNP would report greater levels of emotional distress 1 year post-injury, as measured by the total score on the Hospital Anxiety and Depression Scale (HADS).
2. The association between COMT Val158 and emotional distress would be moderated by age, sex, and injury severity. Based on the literature,^36^ we hypothesized that the COMT Val158 SNP would be most strongly associated with higher levels of emotional distress among female participants, older adult participants, and participants with a longer duration of post-traumatic amnesia (PTA).
3. Adults with moderate-severe TBI carrying one or more copies of the BDNF 66Met SNP would report greater levels of emotional distress 1 year post-injury, as measured by the total score on the HADS.
4. The association between BDNF 66Met and emotional distress would be moderated by age, sex, and injury severity. Based on the literature,^36^ we hypothesized that the BDNF 66Met SNP would be most strongly associated with higher levels of emotional distress among younger adult participants, and participants with a longer duration of PTA. No specific direction was hypothesised regarding an interaction between BDNF 66Met and sex on emotional distress, due to mixed findings in the extant literature.

## Methods

### Participants

Participants in this study were sampled from the Longitudinal Head Injury Outcome Study (LHS) at the Monash-Epworth Rehabilitation Research Centre (MERRC). Written consent was collected from all participants or their next of kin during their initial inpatient stay. Ethics approval was obtained from the Monash University Human Research Ethics Committee.

Data were obtained from consecutive inpatient TBI admissions to Epworth HealthCare (Melbourne, Australia) between 1999 and 2020. Adults (i.e., age >16 years) who had sustained a moderate-severe TBI and completed the 1-year LHS follow-up were included. There were 377 (mean = 40.14 years, 75.4% male) participants who provided COMT genotype data and 303 (mean = 40.65 years, 76.2% male) who provided BDNF genotype data. Participants with any pre- or post-injury neurological condition other than moderate-severe TBI (*n* = 4) or who had missing data at the 1-year LHS follow-up (*n* = 4) were excluded from this study.

### Measures

#### Demographic and injury variables

Demographic and injury-related data were obtained from participant medical records. Injury variables included cause of injury, date of injury, history of previous head injury, and measures of injury severity (Westmead PTA scale,^42^ worst 24-h Glasgow Coma Scale [GCS] score,^43^ and computed tomography [CT] brain result).^43^ All participants in this project had sustained a moderate-severe TBI according to the Mayo classification system (i.e., at least one of: PTA duration ≥1 day, GCS ≤12, and/or acute intracranial abnormality on CT scan).^44^

#### Emotional distress

The Hospital Anxiety and Depression Scale (HADS) was used to assess symptoms of emotional distress during the previous week.^45^ The HADS is a self-report measure with 14 items on a four-point Likert scale (some items are reverse scored). The measure excludes many symptoms of anxiety and depression that overlap with symptoms of TBI such as sleep disturbance and cognitive problems. The total score on the HADS, as opposed to the subscale scores, was used as an index of general emotional distress for a few reasons. Psychometric evidence suggests that the total score, measuring general distress, may be more valid than the traditional subscales, which are highly correlated and do not discriminate between formal anxiety and depressive disorders.^34,46^ Further, the one factor model of the HADS yielded a good fit in similar samples to the current project, and has been supported in the literature as a sound general distress measure.^35,37^ We summed scores on all items of the HADS into a total score (range = 0–42), with higher overall scores indicating greater emotional distress.

### Procedure

Participants provided saliva samples for genetic subtyping during their inpatient stay at Epworth HealthCare. Approximately 1 year after TBI, participants completed the HADS as part of the LHS follow-up with a psychologist-researcher over the phone or in person, or on hard copy by mail.

### Genetic analysis

Genomic data (genomic DNA; gDNA) were extracted from participant saliva samples using the ReliaPrep gDNA Tissue Miniprep system (Promega, Madison, WI). Each gDNA sample was genotyped for the COMT Val158Met and BDNF Val66Met SNPs using one-step amplified refractory mutation system (ARMS) polymerase chain reaction (PCR)^47^ to amplify each allele at each locus. ARMS-PCR products were run on 2% agarose gels and visualized using SYBR Safe (Thermo Fisher Scientific, Waltham, MA) staining.

### Statistical analysis

Data analysis was completed using R Studio (version 3.6). Little's test of missing completely at random (MCAR) highlighted that the missing data in this project were MCAR (*p* = 0.65; see Supplementary Text regarding missing data and Supplementary Table S1). Assumptions checks indicated that no data transformations were necessary. Because of the low number of COMT Val158 and BDNF 66Met homozygotes, participants were coded as either carriers (i.e., carrying at least one copy of the risk SNP for each gene) or non-carriers (i.e., carrying no copies of the risk SNP for each gene).

We constructed two linear regression models—one for COMT Val158 and one for BDNF 66Met—to assess the association between each SNP and emotional distress (total HADS score) post-TBI. The predictor variables included in each regression analysis were: COMT Val158 or BDNF 66Met carriage status (carrier vs. non-carrier), age, sex, PTA duration (days), history of a previous head injury before the index TBI (yes/no), and four interaction terms: SNP × age, SNP × sex, SNP × PTA duration (days), and SNP × sex × age. We chose to use PTA duration as our single metric for injury severity in analyses, as it has demonstrated utility in characterising TBI in acute post-injury stages.^33,48–50^

Previous literature has suggested that pre-injury mental health problems are a strong predictor of emotional distress post-TBI,^3,7^ and COMT Val158 and BDNF 66Met SNPs have been associated with emotional distress in persons without a TBI.^51–56^ As such, we assessed whether any associations between the SNPs and post-TBI emotional distress remained after removing participants who had reported pre-injury mental health problems (COMT, *n =* 121; BDNF, *n* = 99). Pre-injury mental health information was obtained from two sources: 1) participant medical records documenting pre-injury treatment for mental health problems; and 2) a psychiatric study in which a subset of our sample (COMT, *n =* 112; BDNF, *n =* 92) completed the Structured Clinical Interview for Diagnostic and Statistical Manual of Mental Disorders, 4th Edition, Text Revision (DSM-IV-TR) Axis I Disorders (SCID).^7^ The SCID was completed by participants early after their injury (on average, 2 months) and assessed the presence of pre-injury Axis I psychiatric disorders.

Participants were coded as having a pre-injury mental health problem (COMT, *n* = 121; BDNF, *n* = 99) if there was documentation of pre-injury treatment for mental health problems recorded in the participant medical records and/or a recorded pre-injury diagnosis of a psychiatric disorder on the SCID interview, if completed (see Supplementary Text for a comprehensive breakdown of the agreement between the participant medical records and the SCID in characterizing participants' pre-injury mental health problems).

In an additional sensitivity analysis, we included year of injury as a covariate in our regression models to examine whether changes in post-TBI treatment and non-treatment-related factors over time may have influenced the results.

## Results

### Sample characteristics

Of the participants who completed the HADS at the 1-year follow-up, genetic data were available for *n* = 391 (COMT) and *n* = 311 (BDNF) participants. Participants who had complete data across all covariates (COMT, *n* = 377; BDNF, *n* = 303) were included in the regression analyses. All participants sustained a moderate-severe TBI (according to at least one injury severity index), most commonly from a vehicular accident. For full sample characteristics, refer to Tables 1 and 2.

**Table 1.** Sample Frequencies of Injury, Demographic, and Categorical Variables for COMT (*n* = 391) and BDNF (*n* = 311) Samples

	COMT sample (*n* = 391)		BDNF sample (*n* = 311)	
Variable	*n*	%	*n*	%
Sex				
Male	295	75.40	237	76.20
Female	96	24.60	74	23.80
Geographical region of birth				
Oceanian	339	86.70	269	86.70
North-West European	24	6.00	19	6.00
South-East European	5	1.30	4	1.30
North African and Middle Eastern	3	0.80	3	0.80
South-East Asia	2	0.50	2	0.50
North-East Asia	1	0.25	1	0.25
Southern-Central Asia	11	2.95	11	2.95
People of the Americas	2	0.50	2	0.50
Sub-Saharan Africa	2	0.50	2	0.50
Not reported	2	0.50	2	0.50
Caucasian				
Yes	370	94.60	294	94.50
No	21	5.40	20	5.50
Injury cause				
Car accident	208	53.30	165	53.30
Pedestrian accident	67	17.10	56	18.00
Motorcycle accident	60	15.30	48	15.40
Bicycle accident	19	4.90	18	5.70
Fall	14	3.60	11	3.60
Sport injury	1	0.30	1	0.30
Work	14	3.60	7	2.20
Assault	1	0.30	1	0.30
Horse accident	2	0.30	1	0.30
Other	5	1.30	3	0.90
Previous TBI				
Yes	11	2.90	8	1.70
No	380	97.10	306	98.30
Pre-injury treatment for mental health problems				
Yes	96	24.60	78	25.10
No	295	75.40	233	74.90
				

COMT, catechol-*O*-methyltransferase; BDNF, brain-derived neurotrophic factor; TBI, traumatic brain injury.

**Table 2.** Means, Standard Deviations, and Correlations Between Continuous Variables

	Variable	M	SD	1	2	3	4
COMT *s*ample (*n* = 391)							
	1. HADS total	12.51	9.05		0.04	0.00	–0.05
	2. Age	40.14	18.11			–0.08	–0.03
	3. PTA duration	20.98	21.76				0.07
	4. Time post-injury	430.28	216.28				
BDNF sample (*n* = 311)							
	1. HADS total	12.31	9.04		0.06	–0.03	–0.05
	2. Age	40.65	18.34			–0.07	–0.08
	3. PTA duration	21.20	22.50				0.10
	4. Time post-injury	419.07	166.81				

^*^ *p* < 0.05.

COMT, catechol-*O*-methyltransferase; BDNF, brain-derived neurotrophic factor; PTA, post-traumatic amnesia; HADS, Hospital Anxiety and Depression Scale; M, mean; SD, standard deviation.

### COMT Val158Met and BDNF Val66Met single-nucleotide polymorphisms

A total of 299 participants (76.4%) of our COMT sample carried at least one copy of the COMT Val158 SNP, and 106 (34%) of our BDNF sample carried at least one copy of the BDNF 66Met SNP (see Table 3). COMT and BDNF SNP frequencies were consistent with the Hardy-Weinberg equilibrium; they did not differ significantly from the frequencies observed in wider Caucasian populations (COMT: *χ^2^* = 0.85, *p* = 0.52; BDNF: *χ^2^* = 0.66, *p* = 0.40). SNP frequencies in this study were also consistent with those reported in previous Caucasian TBI samples.^12,57^ COMT Val158 and BDNF 66Met carriers and non-carriers did not differ significantly across any covariates (see Supplementary Tables S1–S4).

**Table 3.** Participant Genotypes Across the COMT (*n* = 391) and BDNF (*n* = 311) SNPs

Candidate genotype	*n*	%
*COMT Val158* carrier	299	76.50
*Val158/158Met*	207	53.00
*Val158/Val158*	92	23.50
*COMT Val158Me*t non-carrier: *158Met/158Met*	92	23.50
*BDNF 66Met* carrier	106	34.00
*Val66/66Met*	94	30.20
*66Met/66Met*	12	3.80
*BDNF 66Met* non-carrier: *Val66/Val66*	205	66.00

COMT, catechol-*O*-methyltransferase; BDNF, brain-derived neurotrophic factor; SNPs, single-nucleotide polymorphisms.

### COMT Val158 single-nucleotide polymorphism and emotional distress

**FIG 1.**
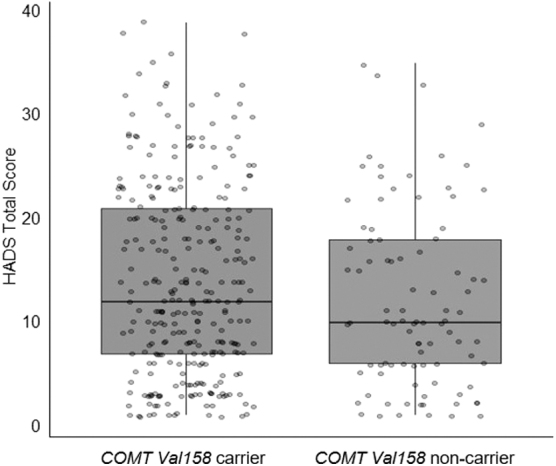
Box-plot comparison of HADS scores between COMT Val158 carriers (*n* = 299) and non-carriers (*n* = 92). In multiple linear regression, there was no significant difference in reported levels of emotional distress between carriers and non-carriers 1 year after moderate-severe TBI. COMT, catechol-*O*-methyltransferase; HADS, Hospital Anxiety and Depression Scale; TBI, traumatic brain injury.

There was no significant main effect of the COMT Val158 SNP on emotional distress (*p* = 0.49; see Fig. 1). There were also no significant interactions between COMT Val158 and sex (*p* = 0.09), COMT Val158 and age (*p* = 0.90), COMT Val158 and PTA duration (*p* = 0.53), or COMT Val158, sex, and age (*p* = 0.08). Full regression output is provided in Table 4.

**Table 4.** Coefficients, Confidence Intervals, and *p* Values in Multiple Linear Regression Predicting Emotional Distress for COMT (*n* = 377) and BDNF (*n* = 303)

Variable	β	$\eta_{p}^{2}$	95% CI	*p *value
*COMT* regression				
*COMT Val158*	–2.30	<0.001	[−8.89, 4.28]	0.49
Age at assessment	0.04	<0.001	[−0.03, 0.11]	0.30
Sex	5.25	0.01	[−1.10, 11.61]	0.10
PTA duration (days)	–0.003	<0.001	[−0.05, 0.05]	0.88
Previous head injury	5.70	0.01	[−0.05, 11.43]	0.05
*COMT* × age	–0.01	<0.001	[−0.17, 0.15]	0.90
*COMT* × sex	–10.27	0.01	[−22.03, 1.49]	0.09
*COMT* × PTA duration	0.03	<0.001	[−0.06, 0.13]	0.53
*COMT* × sex × age	0.23	0.01	[−0.03, 0.49]	0.08
*BDNF* regression				
*BDNF 66Met*	–1.50	<0.001	[−8.13, 5.12]	0.66
Age at assessment	0.05	<0.001	[−0.07, 0.16]	0.41
Sex	0.96	<0.001	[−10.76, 12.68]	0.87
PTA duration (days)	–0.02	<0.001	[−0.11, 0.07]	0.66
Previous head injury	2.80	<0.001	[−3.72, 9.33]	0.40
*BDNF* × age	–0.006	<0.001	[−0.15, 0.14]	0.93
*BDNF* × sex	3.70	<0.001	[−10.13, 17.52]	0.60
*BDNF* × PTA duration	0.008	<0.001	[−0.10, 0.11]	0.87
*BDNF* × sex × age	–0.05	<0.001	[−0.34, 0.25]	0.76

“β” represents the regression standardized beta coefficient. $\eta_{p}^{2}$ represents the variance explained by a given variable of the total variance, after accounting for variance explained by other variables in the model. The 95% CI is the 95% confidence interval for the standardized beta coefficient.

COMT, catechol-*O*-methyltransferase; BDNF, brain-derived neurotrophic factor; PTA, post-traumatic amnesia; TBI, traumatic brain injury.

There was a significant main effect of previous head injury (*p* = 0.05), whereby persons who had sustained a head injury before their index TBI reported greater emotional distress than those who had no past head injuries. The effect size was small, accounting for 1% of the variance in reported emotional distress. On the other hand, the main effects of sex (*p* = 0.10), age (*p* = 0.30), and PTA duration (*p* = 0.88) were non-significant.

### BDNF 66Met single-nucleotide polymorphism and emotional distress

**FIG 2.**
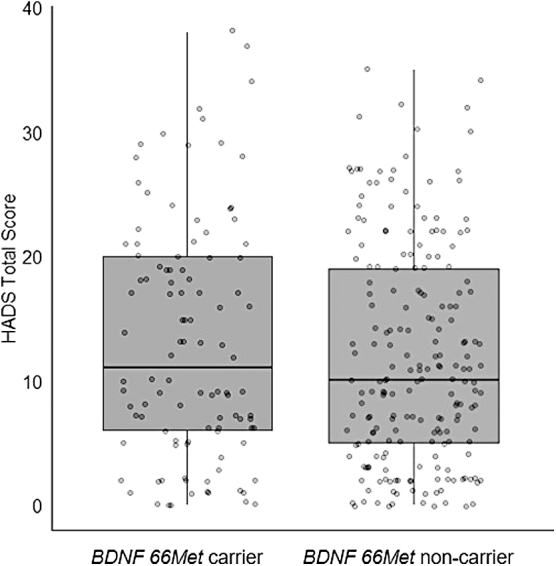
Box-plot comparison of HADS scores between BDNF 66Met carriers (*n* = 106) and non-carriers (*n* = 205). In multiple linear regression, there was no significant difference in reported levels of emotional distress between carriers and non-carriers 1 year after moderate-severe TBI. BDNF, brain-derived neurotrophic factor; HADS, Hospital Anxiety and Depression Scale; TBI, traumatic brain injury.

Similarly, there was no significant main effect of BDNF Val66Met genotype on emotional distress (*p* = 0.66; see Fig. 2). There were also no significant interactions between BDNF 66Met and sex (*p* = 0.60), BDNF 66Met and age (*p* = 0.93), BDNF 66Met and PTA duration (*p* = 0.87), or BDNF 66Met, sex, and age (*p* = 0.76). Full regression output is provided in Table 4.

The main effects of sex (*p* = 0.87), age (*p* = 0.41), PTA duration (*p* = 0.66), and previous head injury (*p* = 0.40) were non-significant.

#### Influence of pre-injury mental health and year of injury

Complete data from only those participants who had not reported pre-injury mental health problems (COMT, *n* = 256; BDNF, *n =* 204) were included in the sensitivity analyses. The pattern of results was the same when analyzing only those persons without pre-injury mental health problems. Full regression output for this sensitivity analysis is provided in Supplementary Table S5.

In an additional sensitivity analysis, the results were also the same when including year of injury as a covariate. There was one exception, which was that the main effect of previous head injury was no longer significant in the regression analysis focusing on COMT (*p* = 0.70). Full regression output for this sensitivity analysis is provided in Supplementary Table S6.

## Discussion

We investigated whether the COMT Val158 and BDNF 66Met SNPs were associated with emotional distress 1 year after moderate-severe TBI. Additionally, we explored whether any associations between these SNPs and emotional distress were moderated by age, sex, and injury severity. Contrary to our hypotheses, we did not observe any significant associations between the SNPs and emotional distress, nor did we find significant interactions of these SNPs with age, sex, or injury severity. The main effects of the covariates were all non-significant, except that, in the regression analyzing COMT, persons with a head injury before the index TBI reported greater emotional distress. In our sensitivity analyses, our null genetic associations persisted when only considering participants without pre-injury mental health problems and when accounting for year of injury.

### No associations of COMT Val158 and BDNF 66Met single-nucleotide poilymorphisms with emotional distress after moderate-severe traumatic brain injury

Contrary to some previous studies, we did not find the COMT Val158 or BDNF 66Met SNPs to be predictive of emotional distress 1 year after moderate-severe TBI. Though our model did account for potential moderating factors of age, sex, and TBI severity, there are other important factors that may have impacted the associations of the COMT Val158 and BDNF 66Met SNPs with emotional distress. For instance, our prediction model for COMT did not account for individual differences in dopaminergic system disruption post-TBI. There is large heterogeneity in the extent to which dopaminergic disruption occurs between persons after TBI.^58^ As such, the association of the COMT Val158 SNP with post-injury outcomes may vary depending on the extent of dopaminergic system changes. Heterogeneity in dopaminergic recovery could be attributable to a number of factors, including the brain regions damaged in the injury and the person's pre-injury dopaminergic system function.^58–60^

Regarding the null BDNF findings, there is evidence to suggest that BDNF is sensitive to life stress exposure, and we did not account for this environmental factor. BDNF proteins play a role in stress hormone signaling pathways, and stress exposure can result in a reduction in BDNF protein concentration. In turn, the BDNF Val66Met genotype, which also influences the concentration of BDNF proteins, may be related to experiences of emotional distress in the face of life stressors.^61^ Indeed, one meta-analysis found that the relationship between exposure to life stressors and depression was stronger among persons with the BDNF 66Met SNP.^62^ Thus, in the current study, the BDNF 66Met SNP in isolation may not have had sufficient power to significantly predict post-TBI emotional distress, given that other environmental factors may have also been driving changes in BDNF protein concentration after injury.

Further, we did not account for variation in other genetic factors that may be related to emotional distress post-TBI. Previous studies, including a recent study from this group, have reported significant associations between other candidate SNPs, such as APOE ɛ4, and emotional distress after TBI.^37,59,63^ Previous findings, taken together with the null associations found in this study, suggest that candidate SNPs in isolation may contribute modestly to emotional distress after TBI. It is therefore recommended that future studies in this area move toward integrating multiple genetic factors, and their potential moderating factors, in predicting complex polygenic traits such as emotional distress after TBI.^64^

### Study strengths and limitations

There are several limitations to the current study that should be considered. First, despite the sample sizes in this study being larger than those in many previous studies, they may not have been large enough to detect small, modest associations of single candidate genes on a complex polygenic trait such as emotional distress. Replication of this study in larger, independent samples would be helpful to confirm our null findings. Second, although the HADS has been validated as a measure of emotional distress in TBI populations, the combined total score integrates heterogenous symptoms of anxiety and depression that may show differential associations with genetic factors.^65,66^ Future studies could explore the associations between genetic factors and more narrowly defined symptom dimensions of emotional distress (e.g., anhedonia, hyperarousal).^67^

Additionally, participants in this study all received rehabilitation through a no-fault accident compensation system, which may have influenced their level of emotional distress at the time of assessment. Fourth, our sample was predominantly from Oceanian and European regions, potentially limiting the generalizability of our findings given that gene-outcome associations may be influenced by race.^36^ Racial or ethnic data were not collected in this study, therefore, our estimate for the number of Caucasian participants was indexed from their reported geographical region of birth. The lack of available ancestry data in this study is important to consider, because previous literature has demonstrated that race may influence emotional outcomes in moderate-severe TBI survivors. Moderate-severe TBI survivors from culturally and linguistically diverse (CALD) backgrounds have reported to experience worse depression and anxiety in the first 2 years post-injury compared to non-CALD groups.^68^ It is unclear whether candidate SNPs play a role in this discrepancy. Therefore, future studies would benefit from more accurate characterization of their sample's racial/ethnic background. Fifth, while the Mayo Classification is a broad indicator of injury severity with relative accuracy, all injury severity metrics lack precision due to the many factors that impact outcomes.^44^

Last, having two sources of pre-injury mental health data (i.e., medical records and SCID data) may be considered a limitation of this study. Medical records alone may not have captured pre-injury mental health data accurately for participants who did not also complete a comprehensive psychiatric interview such as the SCID. Future studies should consider using a singular, comprehensive measure of pre-injury mental health.

Nonetheless, our study has key strengths. This is one of the largest studies to date investigating associations of the COMT Val158 and BDNF 66Met SNPs with emotional distress after TBI. It is the first study to consider the influence of moderating factors on these gene-outcome associations. Incorporating moderating factors into gene-outcome association studies allows researchers to consider whether the contribution of genetic factors to outcomes vary across particular characteristics of the person, which can lead to more finely tuned models of genetic risk. Finally, we believe it is valuable to report null findings from well-conducted studies. The reporting of null findings such as ours is important to avoid publication bias and prompt researchers to consider the challenges of single-gene candidate studies in understanding post-TBI outcomes. Our findings prompted us to consider what else could be missing from our analyses (e.g., individual differences in dopaminergic function and life stress exposure) and also suggest that the genetic underpinnings of emotional distress post-TBI will be better understood through polygenic models, whereby multiple SNPs (such as COMT Val158, BDNF 66Met, and APOE ɛ4) may influence emotional distress additively or synergistically.^64^ Further, future studies could consider including gene-gene interactions in their polygenic models, as candidate SNPs such as COMT Val158 BDNF 66Met may only exert their influence on emotional distress post-injury, in the presence of other genetic factors (e.g., APOE ɛ4).

## Conclusion

This study reports null results suggesting that COMT Val158 and BDNF 66Met SNPs do not aid prediction of emotional distress 1 year after moderate-severe TBI, neither in isolation nor in interaction with age, sex, or injury severity. Independent replication of this study in larger samples will provide further clarity as to whether the COMT Val158 and BDNF 66Met SNPs are important for emotional distress after TBI. Our findings highlight the importance of reporting null findings in the TBI gene-outcome literature, given that they may prompt researchers to re-evaluate the strength of previous analyses and evaluate the utility of single-candidate gene approaches in understanding complex polygenic traits. Future analyses in large samples that incorporate multiple genetic (e.g., using polygenic risk scores) and moderating factors (e.g., age, sex, TBI severity, dopaminergic function, and stressful life events) may provide a greater understanding than single-gene studies of the role of genetics in post-TBI emotional distress.

## Transparency, Rigor, and Reproducibility Summary

This project was completed by the authors at MERRC. The data included in this study were part of the 1-year follow-up of the Longitudinal Head Injury Outcomes Study being conducted at MERRC. Participants were recruited into this longitudinal study from consecutive inpatient TBI admissions to Epworth HealthCare, a rehabilitation hospital in Melbourne, Australia. The authors of this project received ethics approval from the Monash University Human Ethics Research Committee and Epworth HealthCare. Genetic subtyping of the COMT Val158 and BDNF 66Met SNPs for each participant was completed by Dr. Richard Burke of Monash University (School of Biological Sciences).

The analyses completed in this study were conducted in conjunction with a sample of moderate-severe TBI survivors who provided APOE ɛ4 genetic data, as part of the wider Longitudinal Head Injury Outcomes Study being conducted at MERRC.^35^ All participant data, including genetic and injury-related data, were kept deidentified to maintain confidentiality and privacy. Data organization and statistical analyses were conducted using R Studio (version 3.6). Data analyses were completed for 391 participants with COMT data, and 311 participants with BDNF data, and included reviewing medical files in an online database for participant information, including emotional distress scores, demographic data, and injury-related variables.

Emotional distress was measured as the total score on the HADS, a previously validated measure of emotional distress after TBI. Because of the low number of COMT Val158 and BDNF 66Met homozygotes in the samples, COMT and BDNF genotypes were dichotomized as Val158 and 66Met carriers (at least one copy of the risk SNP) versus non-carriers (no copy of the risk SNP). Multiple linear regression analyses were conducted to investigate the association between COMT Val158 and BDNF 66Met and emotional distress. Other variables included in the analyses were age, sex, PTA duration (days), previous head injury (yes/no), and four interaction terms (SNP × age, SNP × sex, SNP × age × sex, and SNP × TBI severity). Our selection of variables to include in the model and hypotheses was guided by the previous literature. A sensitivity analysis was completed, whereby the multiple linear regression was rerun using only participant data from persons who were not indicated to have had pre-injury mental health problems. The threshold for statistical significance was set at *p* < 0.05. In an additional sensitivity analysis, we included year of injury as a covariate in our regression models to examine whether changes in post-TBI treatment and non-treatment-related factors over time may have influenced the results. The threshold for statistical significance was set a *p* < 0.05.

Inspection of density and Q-Q plots suggested that the distribution of HADS total scores was slightly positively skewed. Both multiple linear regression and negative binomial regression were performed to examine the effect of this positive skew. The pattern of findings did not differ between the two types of regression, suggesting that the positive skew of the HADS data did not impact the results. As such, the output from the multiple linear regression in this article is reported. There were no instances of multi-collinearity between continuous predictors (i.e., all Spearman's *r* < 0.50 and variance inflation factors <10) or categorical predictors (i.e., non-significant chi-square tests of independence). The residual versus predicted scatter plots indicated that most scores centered around zero, with quantile regression lines appropriately parallel to the Lowess smooth line, satisfying the assumption of homogeneity of variance. Each model also met the assumption of independence of errors (i.e., all Durbin-Watson values were ∼2.0).

## Supplementary Material

Supplemental data

Supplemental data

Supplemental data

Supplemental data

## Abbreviations Used

APOE: apolipoprotein E
ARMS: amplified refractory mutation system
BDNF: brain-derived neurotrophic factor
CALD: culturally and linguistically diverse
COMT: catechol-*O*-methyltransferase
CT: computed tomography
GCS: Glasgow Coma Scale
DSM-IV-TR: Diagnostic and Statistical Manual of Mental Disorders, 4th Edition, Text Revision
gDNA: genomic DNA
HADS: Hospital Anxiety and Depression Scale
LHS: Longitudinal Head Injury Outcome Study
MCAR: missing completely at random
MERRC: Monash-Epworth Rehabilitation Research Centre
PCR: polymerase chain reaction
PTA: post-traumatic amnesia
SCID: Structured Clinical Interview for DSM-IV
SNPs: single-nucleotide polymorphisms
TBI: traumatic brain injury
