# Supplementary material for: COMT Val158Met and BDNF Val66Met Single-Nucleotide Polymorphisms Are Not Associated With Emotional Distress One Year After Moderate-Severe Traumatic Brain Injury
Source: Neurotrauma Rep. 2023 Aug 7;4(1):495–506. doi: 10.1089/neur.2023.0028 (PMC10457651; doi:10.1089/neur.2023.0028)
Supplement: Supplemental data [file Suppl_Data.docx]

**Missing Data**

There were four participants who were excluded from this study for incomplete data on the HADS, but who did have genetic data for their COMT (*n* = 4) genotype. All participants who provided BDNF genotype data also completed the HADS. We conducted a Littles’ Test of Missing Completely at Random (MCAR) to assess the pattern of missingness in these four individuals.

**Table S1**

*Littles’ Missing Completely at Random (MCAR) test results*

| *χ²* |  | Degrees Freedom | *p*-value | | Missing patterns |
| --- | --- | --- | --- | --- | --- |
| 2.43 |  | 4 | 0.65 |  | 2 |

**Disagreement between Sources of Pre-Injury Mental Health Data**

There was some disagreement between the two sources of pre-injury mental health data due to being collected by different sources for some participants (i.e., medical records and or SCID data). Of the 121 participants in our *COMT* sample who had both sources of pre-injury mental health data, there were 33 participants (27%) who reported a pre-injury mental health problem in their SCID assessment but had no pre-injury mental health data in their medical records. The most common mental health problems reported in the SCID for these participants were substance abuse and addiction (*n* = 20), depression disorders (*n* = 9), anxiety disorders (*n* = 12) and post-traumatic stress disorders (*n =* 3). There was also one participant who had no pre-mental health problems recorded in the SCID, but a treatment record in their medical record. These individuals were all coded as having had a pre-injury mental health problem. Two participants had missing data for the SCID, but treatment recorded in their medical records, and were therefore coded as having had a pre-injury mental health problem.

Of the 92 participants in our *BDNF* sample who had both sources of pre-injury mental health, there were 26 participants (28%) who reported a pre-injury mental health problem in their SCID assessment but had no pre-injury mental health data in their medical records. The most common mental health problems reported in the SCID for these participants were substance abuse and addiction (*n* = 18), depression disorders (*n* **=** 7**)**, anxiety disorders (*n* = 11) and post-traumatic stress disorders (*n* ***=*** 3**)**. These individuals were all coded as having had a pre-injury mental health problem. One participant had missing data for the SCID, but treatment recorded in their medical records, and was therefore coded as having had a pre-injury mental health problem.
