# Supplementary material for: COMT Val158Met and BDNF Val66Met Single-Nucleotide Polymorphisms Are Not Associated With Emotional Distress One Year After Moderate-Severe Traumatic Brain Injury
Source: Neurotrauma Rep. 2023 Aug 7;4(1):495–506. doi: 10.1089/neur.2023.0028 (PMC10457651; doi:10.1089/neur.2023.0028)
Supplement: Supplemental data [file Suppl_TableS1-S4.docx]

**Table S1**

*T-test Results Comparing COMT 158Val Carriers and Non-carriers Across Continuous Variables*

|  | *Carriers* | | *Non-carrier* | |  |  |  |
| --- | --- | --- | --- | --- | --- | --- | --- |
| *Variable* | *M* | *SD* | M | *SD* | *t* | *p*-value | 95%CI |
| HADS total | 12.97 | 8.95 | 11.03 | 8.56 | 1.81 | 0.06 | [-0.11, 3.99] |
| Age | 40.30 | 17.88 | 39.65 | 18.9 | 0.28 | 0.77 | [-3.76, 5.04] |
| PTA duration | 19.90 | 21.79 | 24.51 | 23.71 | 1.64 | 0.10 | [-10.12, 0.92] |
| Time since injury | 426.65 | 226.3 | 444.16 | 278.55 | -0.56 | 0.58 | [-79.53, 44.52] |
|  |  |  |  |  |  |  |  |

**Table S2**

*T-test Results for Comparing BDNF 66Met and Carriers Across Continuous Variables*

|  | *Carriers* | | *Non-carrier* | |  |  |  |
| --- | --- | --- | --- | --- | --- | --- | --- |
| *Variable* | *M* | *SD* | M | *SD* | *t* | *p*-value | 95%CI |
| HADS total | 13.11 | 9.55 | 11.89 | 8.75 | 1.86 | 0.06 | [-0.11, 3.99] |
| Age | 41.07 | 17.88 | 40.44 | 18.60 | 0.28 | 0.77 | [-3.76, 5.04] |
| PTA duration | 20.73 | 20.59 | 21.45 | 23.50 | -1.67 | 0.10 | [19.90, 24.51] |
| Time since injury | 419.49 | 183.84 | 418.86 | 157.72 | -0.56 | 0.58 | [426.66, 444.164] |

**Table S3**

*Chi-square Test Results Comparing COMT 158Val Carriers and Non-carriers Across Categorical Variables of Sex, Previous TBI and Pre-injury Mental Health Problems*

|  |  | *Carrier* | *Non-carrier* | |  |  |
| --- | --- | --- | --- | --- | --- | --- |
| *Variable* |  | *n* | *n* |  | *χ²* | *p-*value |
| Sex |  |  |  |  |  |  |
|  | Male | 229 | 63 |  | 2.70 | 0.10 |
|  | Female | 66 | 29 |  |  |  |
|  |  |  |  |  |  |  |
| Previous TBI |  |  |  |  |  |  |
|  | Yes | 288 | 7 |  | 0.004 | 0.83 |
|  | No | 88 | 4 |  |  |  |
| Pre-injury mental health problems |  |  |  |  |  |  |
|  | Yes | 100 | 70 |  | 0.40 | 0.52 |
|  | No | 195 | 22 |  |  |  |
|  | **p*<.05 |  |  |  |  |  |

**Table S4**

*Chi-square Test Results Comparing BDNF 66Met Carriers and Non-carriers Across Categorical Variables of Sex, Previous TBI and Pre-injury Mental Health Problems*

|  |  | *Carrier* | *Non-carrier* | |  |  |
| --- | --- | --- | --- | --- | --- | --- |
| *Variable* |  | *n* | *n* |  | *χ²* | *p-*value |
| Sex |  |  |  |  |  |  |
|  | Male | 82 | 155 |  | 0.86 | 0.35 |
|  | Female | 24 | 50 |  |  |  |
|  |  |  |  |  |  |  |
| Previous TBI |  |  |  |  |  |  |
|  | Yes | 1 | 7 |  | 2.79 | 0.09 |
|  | No | 155 | 198 |  |  |  |
| Pre-injury mental health problems |  |  |  |  |  |  |
|  | Yes | 38 | 63 |  | 0.61 | 0.43 |
|  | No | 68 | 142 |  |  |  |
|  | **p*<.05 |  |  |  |  |  |
