# Supplementary material for: COMT Val158Met and BDNF Val66Met Single-Nucleotide Polymorphisms Are Not Associated With Emotional Distress One Year After Moderate-Severe Traumatic Brain Injury
Source: Neurotrauma Rep. 2023 Aug 7;4(1):495–506. doi: 10.1089/neur.2023.0028 (PMC10457651; doi:10.1089/neur.2023.0028)
Supplement: Supplemental data [file Suppl_TableS5.docx]

**Table S5**

*Coefficients, Confidence Intervals and p-values in Multiple Linear Regression Predicting Emotional Distress for COMT (n = 256) and BDNF (n = 204) Participants who had not had Pre-injury Mental Health Problems*

| Variable | *β* | $\eta_{p}^{2}$ | 95%CI | *p*-value |
| --- | --- | --- | --- | --- |
| *COMT* Regression |  |  |  |  |
| *COMT Val158* | 1.77 | <0.001 | [-5.63, 9.18] | 0.63 |
| Sex | 9.18 | 0.02 | [-0.09, 15.93] | 0.06 |
| Age at assessment | 0.07 | 0.01 | [0.00, 0.16] | 0.06 |
| PTA duration (days) | 0.01 | <0.001 | [-0.05, 0.07] | 0.06 |
| Previous head injury | 6.39 | 0.01 | [-0.78, 13.57] | 0.70 |
| *COMT* × age | -12.55 | 0.01 | [-25.52, 0.41] | 0.08 |
| *COMT* × sex | -0.05 | <0.001 | [-0.22, 0.12] | 0.06 |
| *COMT* × PTA duration | -0.02 | <0.001 | -0.13, 0.08] | 0.54 |
| *COMT* × sex × age | 0.23 | 0.01 | [-0.04, 0.52] | 0.09 |
| *BDNF* Regression |  |  |  |  |
| *BDNF 66Met* | -2.56 | <0.001 | [-10.80, 5.68] | 0.54 |
| Sex | -0.33 | <0.001 | [-13.59, 12.92] | 0.96 |
| Age at assessment | 0.07 | 0.01 | -0.07, 0.21] | 0.30 |
| PTA duration (days) | -0.02 | <0.001 | [-0.16, 0.10] | 0.66 |
| Previous head injury | 3.51 | <0.001 | -4.68, 11.70] | 0.39 |
| *BDNF* × age | 5.50 | <0.001 | [-10.49, 21.50] | 0.50 |
| *BDNF* × sex | -0.006 | <0.001 | [-0.18, 0.16] | 0.93 |
| *BDNF* × PTA duration | 0.02 | <0.001 | [-0.12, 0.16] | 0.73 |
| *BDNF* × sex × age | -0.04 | <0.001 | [-0.37, 0.29] | 0.79 |
