# Supplementary material for: COMT Val158Met and BDNF Val66Met Single-Nucleotide Polymorphisms Are Not Associated With Emotional Distress One Year After Moderate-Severe Traumatic Brain Injury
Source: Neurotrauma Rep. 2023 Aug 7;4(1):495–506. doi: 10.1089/neur.2023.0028 (PMC10457651; doi:10.1089/neur.2023.0028)
Supplement: Supplemental data [file Suppl_TableS6.docx]

**Table S6**

*Coefficients, Confidence Intervals and p-values in Multiple Linear Regression Predicting Emotional Distress for COMT (n = 376) and BDNF (n = 301), with Year of Injury included as a Covariate*

| Variable | *β* | $\eta_{p}^{2}$ | 95%CI | *p*-value |
| --- | --- | --- | --- | --- |
| *COMT* Regression |  |  |  |  |
| *COMT Val158* | -2.40 | <0.001 | [-8.98, 4.17] | 0.47 |
| Sex | 5.24 | 0.01 | [-1.10, 11.59] | 0.10 |
| Age at assessment | 0.03 | <0.001 | [-0.03, 0.11] | 0.26 |
| PTA duration (days) | -0.001 | <0.001 | [-0.05, 0.05] | 0.96 |
| Previous head injury | 5.54 | 0.01 | [-0.20, 11.27] | 0.06 |
| Year of injury | -0.15 | <0.001 | [-0.36, 0.05] | 0.03 |
| *COMT* × age | -0.007 | 0.01 | [-0.16, 0.15] | 0.92 |
| *COMT* × sex | -10.26 | 0.01 | [-22.00, 1.48] | 0.08 |
| *COMT* × PTA duration | 0.02 | <0.001 | [-0.07, 0.12] | 0.55 |
| *COMT* × sex × age | 0.23 | 0.01 | [-0.03, 0.49] | 0.88 |
| *BDNF* Regression |  |  |  |  |
| *BDNF 66Met* | -1.44 |  | [-8.08, 5.20] | 0.66 |
| Sex | 1.06 |  | [-10.68, 12.80] | 0.86 |
| Age at assessment | 0.05 |  | [-0.07, 0.16] | 0.41 |
| PTA duration (days) | -0.02 | <0.001 | [-0.11, 0.07] | 0.67 |
| Previous head injury | 2.778 | <0.001 | [-3.76, 9.30] | 0.40 |
| Year of injury | -0.07 | <0.001 | [-0.32, 0.18] | 0.60 |
| *BDNF* × age | -0.01 | <0.001 | [-0.15, 0.4] | 0.93 |
| *BDNF* × sex | 3.63 | <0.001 | [-10.21, 17.48] | 0.61 |
| *BDNF* × PTA duration | 0.01 | <0.001 | [-0.10, 0.11] | 0.87 |
| *BDNF* × sex × age | -0.04 | <0.001 | [-0.34, 0.25] | 0.80 |
